# Genetic Divergence in Domestic Japanese Quail Inferred from Mitochondrial DNA D-Loop and Microsatellite Markers

**DOI:** 10.1371/journal.pone.0169978

**Published:** 2017-01-20

**Authors:** Mitsuo Nunome, Mikiharu Nakano, Ryo Tadano, Ryoka Kawahara-Miki, Tomohiro Kono, Shinji Takahashi, Takaharu Kawashima, Akira Fujiwara, Keijiro Nirasawa, Makoto Mizutani, Yoichi Matsuda

**Affiliations:** 1 Avian Bioscience Research Center, Graduate School of Bioagricultural Sciences, Nagoya University, Nagoya, Japan; 2 Faculty of Applied Biological Sciences, Gifu University, Gifu, Japan; 3 Genome Research Center, NODAI Research Institute, Tokyo University of Agriculture, Tokyo, Japan; 4 Department of Bioscience, Tokyo University of Agriculture, Tokyo, Japan; 5 General Affairs Department, National Institute for Environmental Studies, Tsukuba, Japan; 6 Center for Environmental Biology and Ecosystem Studies, National Institute for Environmental Studies, Tsukuba 305–8506, Japan; 7 Laboratory Animal Research Station, Nippon Institute for Biological Science, Hokuto, Japan; 8 Animal Breeding and Reproduction Research Division, NARO Institute of Livestock and Grassland Science, Tsukuba, Japan; 9 Laboratory of Animal Genetics, Graduate School of Bioagricultural Sciences, Nagoya University, Nagoya, Japan; National Cheng Kung University, TAIWAN

## Abstract

To assess the genetic diversity of domestic Japanese quail (*Coturnix japonica*) populations, and their genetic relationships, we examined mitochondrial DNA (mtDNA) D-loop sequences and microsatellite markers for 19 Japanese quail populations. The populations included nine laboratory lines established in Japan (LWC, Quv, RWN, WE, AWE, AMRP, rb-TKP, NIES-L, and W), six meat-type quail lines reimported from Western countries (JD, JW, Estonia, NIES-Br, NIES-Fr, and NIES-Hn), one commercial population in Japan, and three wild quail populations collected from three Asian areas. The phylogenetic tree of mtDNA D-loop sequences revealed two distinct haplotype groups, Dloop-Group1 and Dloop-Group2. Dloop-Group1 included a dominant haplotype representing most of the quail populations, including wild quail. Dloop-Group2 was composed of minor haplotypes found in several laboratory lines, two meat-type lines, and a few individuals in commercial and wild quail populations. Taking the breeding histories of domestic populations into consideration, these results suggest that domestic quail populations may have derived from two sources, i.e., domestic populations established before and after World War II in Japan. A discriminant analysis of principal components and a Bayesian clustering analysis with microsatellite markers indicated that the domestic populations are clustered into four genetic groups. The two major groups were Microsat-Group1, which contained WE, and four WE-derived laboratory lines (LWC, Quv, RWN, and AWE), and Microsat-Group2 consisting of NIES-L, JD, JW, Estonia, NIES-Br, NIES-Fr, NIES-Hn, W, and commercial and wild populations. The remaining two lines (AMRP and rb-TKP) were each clustered into a separate clade. This hierarchical genetic difference between domestic quail populations is attributed to the genetic background derived from two different genetic sources—the pre-war and post-war populations—which is well supported by their breeding histories.

## Introduction

Japanese quail (*Coturnix japonica*, Phasianidae) is an important animal species in poultry production. Owing to their high growth and egg-laying rates, Japanese quail have been preferentially bred worldwide, including selective breeding to improve egg and meat production, since the beginning of the 20^th^ century [1]. As quail farming has increased, studies have examined the genetic diversity of domestic quail populations as well as physiological and genetic factors affecting economic traits, such as growth rate, egg-laying rate, and disease resistance [1–6]. Japanese quail is also used in several biological studies as a laboratory animal because of its useful biological properties, including a short generation interval and small body size [7,8]. Many laboratory and mutant lines with unique characteristics, such as feather color, egg color, morphology, and hereditary disorders, have been developed [9–11].

Japanese quail were probably domesticated in Japan in the 12^th^ century owing to their pleasant song. Japanese quail were reportedly introduced from Japan to the United States twice, in the 1870s and in the early 20^th^ century, as a game bird [8,12]. Some laboratory lines of Japanese quail are considered to have been established from game bird populations in the United States during the mid-20^th^ century [7,12]. The commercial use of this species for egg and meat production began around 1910 in Japan [10,13] and became active in the 1930s [14]. During this period, Japanese quail was selected for various characteristics, such as body size and plumage color, in Japan [12]. Since the 1930s, domestic quail for egg and meat production were exported from Japan to the United States, Europe, and the Near and Middle East [1,14]. The commercial quail populations in Japan were in danger of extinction during World War II, and were subsequently re-established from a few surviving individuals of commercial quail [13,14], with the probable inclusion of wild Japanese quail, after the end of the war [10,12]. The recovered commercial populations were again exported to foreign countries and were rapidly distributed around the world. Therefore, most domestic Japanese quail that are bred worldwide today are likely descendants of populations that were re-established in Japan after the war [15–17].

Considering the history of domestication of Japanese quail and their distribution around the world, some genetic characteristics of the pre-war population are expected to remain in the present domestic quail populations. Therefore, it is conceivable that domestic Japanese quail populations, including commercial quail and laboratory lines, can be divided into two groups according to origin: one descended from populations distributed before World War II (the pre-war population) and another that was re-established from a limited founder population in Japan after the war (the post-war population). The post-war populations are expected to be closely related at the genetic level owing to the post-war bottleneck and/or founder effect. Population genetic research on domestic quail populations has been performed using protein, mitochondrial DNA, and microsatellite markers [18–24]. Genetic divergence among Japanese quail populations has been studied from a variety of perspectives, e.g., genetic introgression from farm populations of Japanese quail into wild populations of the common quail (*C*. *coturnix*) in European countries [18,19] and genetic differences among wild, commercial, and laboratory Japanese quail populations [20–24]. However, genetic differences between pre-war and post-war quail populations remain unclear.

Mitochondrial DNA (mtDNA) D-loop and microsatellite DNA markers are effective for studying genetic differences among populations within a species because they exhibit a high degree of genetic variability. We recently developed more than 100 microsatellite DNA markers from genomic regions containing 7 to 25 CA repeat elements based on the draft genome sequence of Japanese quail [25], which covered chromosomes 1 to 28 and the Z and W sex chromosomes. Among them, 50 highly variable markers were used to examine the genetic characteristics of 13 domestic populations of Japanese quail in our previous study [24]. In the present study, we characterized 15 laboratory quail lines, including six meat-type quail lines that were reimported from Western countries, one commercial population in Japan, and three wild quail populations collected from Japan and China, by mtDNA D-loop sequencing and microsatellite marker genotyping. We then performed a phylogenetic analysis based on mtDNA D-loop sequences and population genetic analyses based on microsatellite markers. Based on these data, we discuss the involvement of pre-war and post-war populations in the genetic divergence of domestic Japanese quail populations.

## Materials and Methods

### Specimens and genomic DNA extraction

In this study, 19 Japanese quail populations were used, including nine laboratory lines established in Japan, six laboratory lines derived from meat-type quail populations that were reimported from Western countries, one commercial population in Japan, and three wild quail populations collected from three Asian areas, (S1 and S2 Tables, S1 Fig). Genotyping of 50 microsatellite markers has previously been performed for 40 individuals from each of eight laboratory quail lines (LWC, Quv, RWN, WE, AWE, AMRP, rb-TKP, and W), 34 individuals from three meat-type quail lines (six from Estonia, 16 from JD, and 12 from JW), and 12 individuals from a commercial population in our previous studies [24,25]. In this study, the genotypes of 50 microsatellite markers were newly examined for 19 to 21 individuals each from one laboratory line (NIES-L) and three meat-type quail lines (NIES-Br, NIES-Fr, and NIES-Hn), 45 commercial individuals purchased from an animal breeding company in Japan (Motoki Corporation, Saitama, Japan), and 21 wild Japanese quail. These genotype data were incorporated into our published data set [24,25] for the analysis in this study.

Genomic DNA from four laboratory lines (NIES-L, NIES-Br, NIES-Fr, and NIES-Hn) and commercial quail was extracted from 20 μl of blood using 300 μl of DNAzol BD reagent (Molecular Research Center, Tokyo, Japan). Muscle tissues of two wild Japanese quail collected at Tsushima Island were provided by the Yamashina Institute for Ornithology in Japan. The samples were stored in 70% ethanol. Genomic DNA was extracted from tissue samples of about 5 mm^3^ using 30 ng of Proteinase K in 0.7 ml of buffer [50 mM Tris-HCl (pH 8.0), 100 mM NaCl, and 20 mM EDTA], followed by three purification steps using phenol, phenol/chloroform/isoamyl alcohol (25:24:1), and chloroform/isoamyl alcohol (24:1). Feather shaft samples of 19 stuffed specimens of wild Japanese quail, consisting of 14 samples from Honshu of the Japanese archipelago (Nagao, Osaka, Kyoto, Tokyo, and Gunma prefectures) and five samples from the Liaodong Peninsula in China, were provided by three museums, Osaka Museum of Natural History, Gunma Museum of Natural History, and Togakushi Museum of Natural History in Japan. Genomic DNA was extracted from the basal tips of rachises (approximately 1 or 2 mm in length) using commercial DNA extraction kits (DNA Extractor FM Kit, Wako Pure Chemical, Osaka, Japan; QIAmp DNA Investigator Kit, Qiagen, Hilden, Germany).

Animal care and all experimental procedures were approved by the Animal Experiment Committee, Nagoya University (approval no. 2012050101), and were conducted according to the Regulations on Animal Experiments in Nagoya University.

### PCR amplification from feather samples of stuffed specimens

Genomic DNA of historical stuffed specimens is generally degraded and fragmented, and these properties make successful PCR amplification difficult, resulting in the detection of false alleles and/or allelic dropout and leading to genotyping errors [26–28]. To decrease the influence of false alleles due to the failure of PCR amplification, PCR was performed at least three times for each sample. To check for genomic contamination in samples obtained from stuffed specimens, a negative control (non-tissue) sample was prepared for each experiment. The absence of PCR products was checked by 2% agarose gel electrophoresis after PCR.

### Sequencing of mtDNA D-loops and genotyping of microsatellite markers

A 287-bp partial DNA fragment of the mtDNA D-loop region was amplified by PCR using the primer set Quail_D-loop-F (5′-CCTAACTCCCCTACTTAGTGTACC-3′) and Quail_D-loop-R (5′-TCTCGTGAGGTGTACGATCAAT-3′). Amplification was performed in a 15-μl reaction mix containing 50 ng of genomic DNA, 10 pmol each primer, and 7.5 μl of 2 × Phusion HF Master Mix (New England Biolabs, Ipswich, MA, USA). The cycling conditions for PCR were as follows: initial denaturation at 98°C for 30 s, followed by 40 cycles at 98°C for 10 s, 60°C for 15 s, and 72°C for 20 s, and a final extension for 5 min at 72°C. PCR products were purified using the 20% polyethylene glycol/2.5 M NaCl precipitation method [29,30]. The cycle sequencing reaction was performed with the forward primer using a BigDye™ Terminator Cycle Sequencing Kit (ver 3.1, Applied Biosystems, Foster City, CA, USA), and nucleotide sequences were determined using an ABI PRISM 3130 Genetic Analyzer (Applied Biosystems).

PCR amplification of microsatellite markers was performed using a 10-μl reaction mix containing approximately 50 ng of genomic DNA, 10 pmol each primer, and 5.0 μl of Taq Gold 360 Master Mix (Applied Biosystems). The cycling conditions for PCR were as follows: initial denaturation at 95°C for 10 min, followed by 42 cycles at 95°C for 30 s, 55°C for 30 s, and 72°C for 25 s, and a final extension for 5 min at 72°C. The suitable annealing temperature for each primer set was determined in our previous study [24]. PCR products were electrophoresed with Hi-Di formamide (Applied Biosystems) and the GeneScan 600 LIZ Size Standard (Applied Biosystems) using the ABI PRISM 3130 Genetic Analyzer (Applied Biosystems). Allele sizes were determined using GENEMAPPER version 4.1 (Applied Biosystems).

### Data analyses

DNA sequences were aligned using ClustalW [31] implemented in MEGA 6.0 [32] and edited using PROSEQ 2.9.1 [33]. Maximum-likelihood and Bayesian phylogenetic trees were constructed using PhyML 3.0 [34] and BEAST 1.8.3 [35], respectively. The best-fitting substitution models for the maximum-likelihood and Bayesian analyses were determined based on the Bayesian information criterion (BIC) and Akaike information criterion (AIC) using jModeltest 2.1.10 [36]. Branch support was calculated using 1,000 bootstrap replications for the maximum-likelihood analysis. The maximum-likelihood tree was visualized using MEGA 6.0 [32]. The Bayesian analyses were performed using 10 million Markov chain Monte Carlo (MCMC) generations, sampling one tree every 1,000 generations. The convergence of the runs was verified using Tracer 1.6.0 [37]. After removing the first 10% of the sampled 10,000 trees as a burn-in, Bayesian posterior probabilities (BPP) were calculated from the remaining trees using Tree Annotator 1.8.3. FigTree 1.4.2 (http://beast.bio.ed.ac.uk/FigTree) was used to view the Bayesian tree. The following D-loop haplotypes using in previous phylogenetic analyses [18,19] were included: 10 Japanese quail haplotypes found in farm quail and wild-captured quail in Europe [H1 (DQ087957) [18], F1W1 (KF410830) [19], F2−F8 (KF410831‒KF410837) [19], and W6 (KF410842) [19]], one haplotype found in two wild quail in Mongolia [H63 (DQ087958) [18]], and one haplotype of *C*. *coturnix* (KJ623812). The D-loop sequence of *C*. *chinensis* (AB073301) was used as an outgroup taxon. To understand relationships among haplotypes of the D-loop region, a median-joining network [38] was constructed using Network [fluxus-engineering.com].

Based on microsatellite marker data, the mean number of alleles (*MNA*) per locus and expected heterozygosity (*He*) were calculated using ARLEQUIN 3.5.1.2 [39], allelic richness (*AR*) was calculated using MICROSATELLITE ANALYSER 4.05 [40], and the mean number of effective alleles (*Ne*, the number of alleles weighted by the square of the allele frequency) was obtained using GENALEX 6.5 [41]. MICRO-CHECKER 2.2.3 [42] was used to determine null allele markers. The chi-square test for Hardy-Weinberg equilibrium (*HWE*) and calculation of a fixation index (*F*) were carried out for each line and population using GENALEX 6.5 [41]. Pairwise genetic distances among populations (*F*_*ST*_) were calculated using MICROSATELLITE ANALYSER 4.05 [40] with 10,000 permutations to test the level of significance. The exploratory discriminant analysis of principal components (DAPC) [43] was performed using the R package adegenet [44]. The number of PC and clusters were set at 15 and 14, respectively, according to the tutorial document of the analysis. The Bayesian clustering analysis was performed to infer the number of genetic clusters in domestic and wild Japanese quail using STRUCTURE 2.3 [45]. Log probability values (Ln P[D]) were estimated for a broad range of K values (from 2 to 18) with sampling periods of 10,000,000 MCMC generations after burn-in periods of 100,000 generations. The admixture model and correlated allele frequency model were selected for this analysis [46]. Ten independent MCMC runs were performed for each K. The optimal K value was estimated using the Evanno method [47] implemented in STRUCTURE HARVESTER 0.6.94 [48]. MCMC runs were excluded from subsequent analyses as outliers when the variance of Ln likelihood was more than two times higher than that of other MCMC runs within each K (among 10 MCMC runs). The results of the remaining runs for each K were compiled and visualized using CLUMPAK [49], and to infer hierarchical changes in genetic clusters of 19 quail populations, the Bayesian clustering analysis results were compared for K = 2 to K = 17.

## Results

### Haplotypes of the mtDNA D-loop region

The 278-bp nucleotide sequence of the mtDNA D-loop region was successfully determined for 19 Japanese quail populations (accession numbers LC086816‒LC086858, LC149870; S3 Table). Nine haplotypes (JqD−JqD6, JqW1−JqW3) with 12 variable sites, including one indel, six parsimony-informative sites, and five singletons, were obtained (S4 Table). Six haplotypes, JqD1−JqD6, were found for domestic quail, and JqD1 was the prevailing haplotype (Table 1). JqD1 and JqD2 were frequent in wild populations (S2 Table). JqD1 was identical to H1 (DQ087957) and F1W1 (KF410830), the prevailing haplotypes in domestic quail populations in European countries, and JqD2 was identical to F5 (KF410834), the haplotype observed for one farm quail individual in Spain. JqD4 exhibited 99% identity to JqD1. JqD3, JqD5, and JqD6 each showed 99% identity to the haplotype JqD2. The other three haplotypes (JqW1−JqW3) were each found for one individual in the wild populations.

**Table 1:** Haplotypes of the mitochondrial D-loop region in laboratory lines and commercial and wild populations of Japanese quail, GenBank accession numbers of sequences showing the highest identity, and numbers of indels.

Haplotype	Line and population	Most identical sequence in GenBank	Identity (%)	No. of indels (%)
JqD1	LWC, WE, AWE, rb-TKP, NIES-L, JD, JW, NIES-Br, NIES-Hn, W, commercial, wild (Honshu, the Liaodong Peninsula)	DQ087957.1, KF410830	278/278 (100.0)	0/278 (0.0)
JqD2	WE, AWE, AMRP, W, commercial, wild (Tsushima Is., the Liaodong Peninsula)	KF410834	278/278 (100.0)	0/278 (0.0)
JqD3	Quv, RWN, WE, Estonia, NIES-Fr, commercial	KF410834	275/278 (99.0)	0/278 (0.0)
JqD4	commercial (one individual)	KF410830	277/278 (99.6)	0/278 (0.0)
JqD5	RWN (two individuals), W (two individuals)	KF410834	276/278 (99.3)	0/278 (0.0)
JqD6	commercial (one individual)	KF410834	276/278 (99.3)	0/278 (0.0)
JqW1	wild (Honshu)	KF410834	277/279 (99.3)	1/279 (0.3)
JqW2	wild (Honshu)	KF410834	277/278 (99.6)	0/278 (0.0)
JqW3	wild (Tsushima Is.)	KF410834	276/278 (99.3)	0/278 (0.0)

### Phylogenetic relationships among haplotypes of the D-loop region

For phylogenetic analyses, TPM2uf+G and TIM+G were the best-fitting models, according to the BIC and AIC, respectively; however, these models were not implemented in PhyML online 3.0 [34] or BEAST 1.8.3 [35]. The HKY+G and GTR+G substitution models were the fourth- and ninth-highest ranked models according to the BIC and AIC, respectively; however, they were the highest-ranked models among those available in PhyML online 3.0 [34] and BEAST 1.8.3 [35]. We also searched for the best-fitting model using Smart Model Selection implemented in PhyML online 3.0, and the HKY+G+F and GTR+G+F were selected as the best-fitting models according to the BIC and AIC, respectively. Hence, we used the HKY and GTR substitution models with gamma-distributed rates (G) and empirical base frequencies (F) to construct the maximum-likelihood and Bayesian trees.

**Fig 1:**
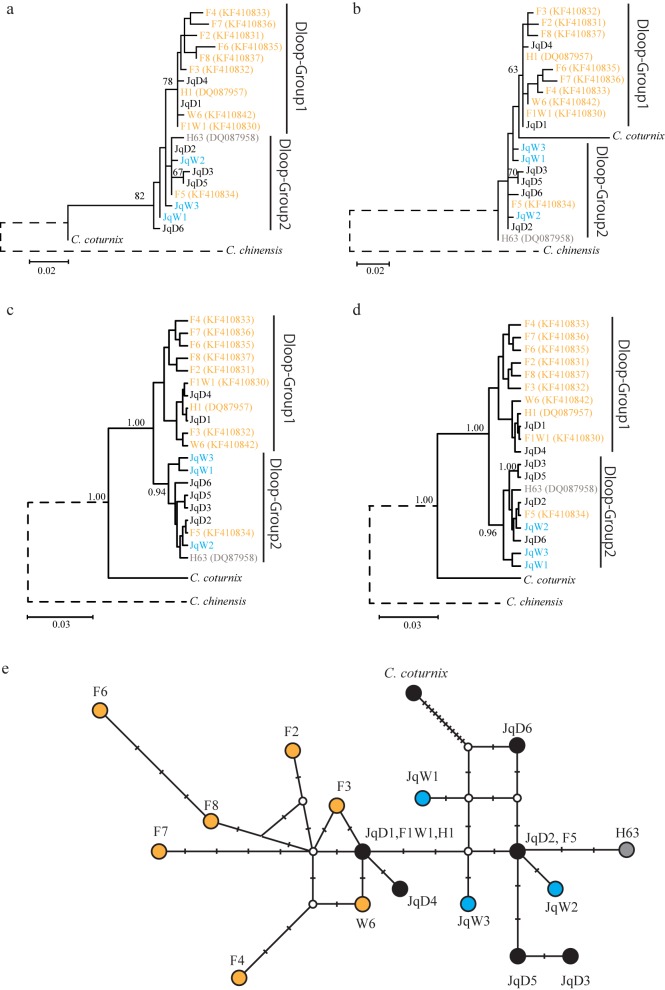
Phylogenetic relationships of mtDNA D-loop haplotypes in domestic and wild populations of Japanese quail. (a−d) Phylogenetic trees constructed using the maximum likelihood method with HKY+G+F (a) and GTR+G+F (b) models and using the Bayesian method with HKY+G+F (c) and GTR+G+F (d) models. Bootstrap values of greater than 50% and Bayesian posterior probabilities of more than 0.90 are shown. JqD1 and JqD4 haplotypes were clustered in the Dloop-Group1 clade with other haplotypes found in domestic Japanese quail in European countries. JqD2, JqD3, JqD5, and JqD6 formed a different clade, Dloop-Group2, with one haplotype (F5) found for one domestic quail in a European farm. JqW1, JqW2, and JqW3 haplotypes from wild quail were closely related to Dloop-Group2 in the Bayesian trees. We shortened the branches of the outgroup species (*C*. *chinensis*, dashed line) because the branch lengths were too long to be shown in the Figure. Haplotypes for domestic and wild-captured quail in European countries, wild quail in Mongolia, and wild quail in this study are indicated in orange, gray, and blue, respectively. (e) Network of mtDNA D-loop haplotypes. JqD2 is closely related to wild Japanese quail haplotypes, JqW1, JqW2, JqW3, and H63, which are distinct from JqD1. Haplotypes found in European farm quail (F2−F4, F6−F8, and W6), except F5, stem from JqD1. The white circle indicates the median vector.

In a phylogenetic analysis, six haplotypes (JqD1−JqD6) were divided into two groups (Fig 1). The maximum likelihood trees showed moderate bootstrap support for one group, Dloop-Group1 (78% for the HKY+G+F model and 63% for the GTR+G+F model) (Fig 1A and 1B), and the Bayesian phylogenetic trees supported the other group, Dloop-Group2, with high posterior probabilities (0.94 for the HKY+G+F model and 0.96 for the GTR+G+F model) (Fig 1C and 1D). Dloop-Group1 included JqD1 and JqD4 found for six laboratory lines in Japan (LWC, WE, AWE, rb-TKP, NIES-L, and W), four meat-type quail lines that originated in the Netherlands, Brazil, and Hungary (JD, JW, NIES-Br, and NIES-Hn), and commercial and wild quail. Dloop-Group1 included nine haplotypes deposited in GenBank for farm quail (H1, F2‒F4, F6‒F8, and F1W1) and wild-captured quail (F1W1 and W6) in Europe.

Dloop-Group2 included four haplotypes, JqD2, JqD3, JqD5, and JqD6, for five laboratory lines (Quv, RWN, WE, AMRP, and W), two meat-type quail lines (Estonia and NIES-Fr), and commercial and wild quail, as well as F5 (KF410834), found for one individual in a farm in Spain. Three unique haplotypes of wild quail (JqW1‒JqW3) and H63 (DQ087958) were contained in Dloop-Group2. In the maximum-likelihood tree based on the GTR+G+F model (Fig 1B), *C*. *coturnix* was positioned between Dloop-Group1 and Dloop-Group2; however, the bootstrap support was very low (<50%). In the network of haplotypes (Fig 1E), JqD1 was the central haplotype of Dloop-Group1. JqD3, JqD5, and JqD6 were more closely related to JqD2 than JqD1. JqW1, JqW2, and JqW3 were genetically more similar to JqD2 than JqD1. All haplotypes of Japanese quail observed in this study were apparently distinct from the haplotype for *C*. *coturnix* (KJ623812).

### Genetic diversity of Japanese quail populations based on microsatellite markers

The numbers of microsatellite markers amplified from each stuffed specimen of wild quail ranged from 5 (A1798 from Honshu) to 20 (A0279 from Honshu) (S1 Table). The total numbers of markers that were amplified from any one individual for each local population were 36 from Honshu, 40 from Tsushima Is, and 23 from the Liaodong Peninsula. PCR amplification of microsatellite markers was difficult for the stuffed specimens of wild individuals. The number of amplified markers from the stuffed specimen did not seem to be correlated with the age of specimens. We could amplify more microsatellite markers from the muscle samples of two wild individuals from Tsushima Is, which were stored in 70% ethanol (S1 Table, and summarized in S2 Fig). Additionally, STRUCTURE analysis assigned three wild quail populations to a single cluster (the details are to be mentioned later). These results suggest that the high frequency of missing data in the stuffed specimens would be caused by the difficulty in PCR amplification with the degraded DNA, not by genetic difference between individuals or technical quality of our analyses. For this reason, we used 23 out of 50 markers that were shared among all three populations for population genetic analyses (S5 Table). Null alleles were frequently found for NGJ0039; however, we treated all 23 markers, including this marker, the same. There were not any populations that showed noticeable deviations from the Hardy-Weinberg equilibrium in 23 microsatellite markers (S6 Table), and all populations, excepting Quv, RWN and rb-TKP, exhibited *F* values close to zero (S6 Table), suggesting little or no genetic subdivision within each populations. The mean number of alleles (*MNA*) ranged from 1.48 to 5.70 for 15 laboratory lines and commercial and wild populations (Table 2). *MNA* was relatively low in five laboratory lines (RWN, AMRP, rb-TKP, Estonia, and NIES-Fr), ranging from 1.48 to 1.65, whereas it was higher in NIES-Br, NIES-Hn, W, and commercial and wild (Honshu) populations, ranging from 2.83 to 5.70. Five lines (RWN, AMRP, rb-TKP, NIES-L, and NIES-Fr) exhibited low expected heterozygosity (*He*) values (0.15 to 0.22); in contrast, high *He* values (0.46 to 0.58) were observed in NIES-Br, W, and commercial and wild (Tsushima Is. and Honshu) populations. The allelic richness (*AR*) was relatively higher for WE, NIES-Br, W, and commercial and wild (Tsushima Is. and Honshu) populations (from 1.68 for WE to 2.03 for the wild population from Honshu) and lower for AMRP, RWN, rb-TKP, Estonia, and NIES-Fr lines (1.22 for rb-TKP to 1.34 for RWN, Estonia, and NIES-Fr). The ranking of these 17 quail populations based on the numbers of effective alleles (*Ne*) was quite similar to the rankings based on *MNA*, *He*, and *AR*. Pairwise *F*_*ST*_ was 0.40, on average, ranging from -0.02 (Honshu and the Liaodong peninsula) to 0.73 (rb-TKP and NIES-Fr), and all *F*_*ST*_ values were significantly greater than zero (P < 0.05, Table 3), excepting values between three wild quail populations. NIES-Br and W lines and the commercial population exhibited relatively small genetic distances to the wild Japanese quail (*F*_*ST*_ = 0.19 to 0.26, 0.21 to 0.33, and 0.17 to 0.18, respectively), whereas RWN, AMRP and rb-TKP exhibited larger genetic distances to the wild Japanese quail (*F*_*ST*_ = 0.54 to 0.66, 0.52 to 0.58 and 0.60 to 0.70, respectively). The *F*_*ST*_ distances between the populations were shown as a neighbor-joining tree using MEGA 6.0 (S3 Fig).

**Table 2:** Genetic diversity of laboratory lines and commercial and wild populations of Japanese quail estimated using 23 microsatellite markers.

Line and population	*N*	*MNA*	*He*	*AR*	*Ne*
LWC^*^	40	2.44	0.36	1.58	1.78
Quv^*^	40	2.35	0.25	1.38	1.49
RWN^*^	40	1.61	0.20	1.34	1.39
WE^*^	40	2.83	0.41	1.68	2.01
AWE^*^	40	2.61	0.42	1.65	2.01
AMRP^*^	40	1.65	0.21	1.32	1.36
rb-TKP^*^	40	1.48	0.15	1.22	1.27
NIES-L	20	2.04	0.22	1.40	1.47
JD^*^	16	2.57	0.37	1.62	1.97
JW^*^	12	2.44	0.40	1.66	1.93
Estonia^*^	6	1.52	0.22	1.34	1.40
NIES-Br	20	3.09	0.46	1.79	2.19
NIES-Fr	19	1.65	0.20	1.34	1.35
NIES-Hn	20	2.70	0.42	1.67	2.02
W^*^	40	3.96	0.55	1.93	2.51
commercial^**^	57	5.70	0.58	2.02	2.96
Tsushima Is.	2	2.30	0.57	2.01	2.11
Honshu	14	3.35	0.57	2.03	2.51
the Liaodong Peninsula	5	1.91	0.42	1.72	1.75

^*^Genotype data were taken from our previous study [24].

^**^Genotype data of 12 individuals out of 57 commercial quail were taken from our previous study [25].

N: Number of individuals.

MNA: Mean number of alleles per locus.

He: Expected heterozygosity.

AR: Allelic richness.

Ne: No. of effective alleles = 1 / (Sum pi^2), where Sum pi^2 is the sum of the squared population allele frequency.

**Table 3:** Pairwise *F*_*ST*_ genetic distances among laboratory lines and commercial and wild populations of Japanese quail.

	LWC	Quv	RWN	WE	AWE	AMRP	rb-TKP	NIES-L	JD	JW	Estonia	NIES-Br	NIES-Fr	NIES-Hn	W	commercial	Tsushima Is.	Honshu	the Liaodong Peninsula
LWC																			
Quv	0.42																		
RWN	0.45	0.52																	
WE	0.21	0.26	0.38																
AWE	0.22	0.30	0.31	0.17															
AMRP	0.49	0.51	0.56	0.38	0.43														
rb-TKP	0.57	0.67	0.65	0.51	0.52	0.66													
NIES-L	0.44	0.63	0.63	0.41	0.42	0.64	0.68												
JD	0.38	0.49	0.51	0.33	0.31	0.50	0.58	0.46											
JW	0.40	0.53	0.55	0.35	0.35	0.52	0.59	0.41	0.12										
Estonia	0.45	0.60	0.64	0.42	0.40	0.60	0.72	0.50	0.46	0.43									
NIES-Br	0.32	0.41	0.43	0.27	0.21	0.44	0.54	0.37	0.18	0.23	0.36								
NIES-Fr	0.51	0.53	0.60	0.38	0.38	0.60	0.73	0.61	0.44	0.46	0.60	0.43							
NIES-Hn	0.38	0.47	0.53	0.32	0.32	0.44	0.55	0.43	0.28	0.23	0.31	0.24	0.43						
W	0.22	0.36	0.38	0.19	0.23	0.39	0.36	0.27	0.21	0.20	0.30	0.18	0.34	0.21					
commercial	0.23	0.29	0.32	0.15	0.18	0.30	0.36	0.28	0.23	0.22	0.26	0.16	0.28	0.18	0.11				
Tsushima Is.	0.40	0.49	0.54	0.31	0.28	0.58	0.68	0.56	0.31	0.32	0.54	0.19	0.54	0.33	0.21	0.17			
Honshu	0.42	0.47	0.56	0.34	0.32	0.52	0.60	0.47	0.32	0.29	0.30	0.25	0.44	0.21	0.21	0.17	0.13^†^		
the Liaodong Peninsula	0.49	0.56	0.66	0.39	0.38	0.58	0.70	0.56	0.38	0.34	0.50	0.26	0.56	0.30	0.26	0.18	0.18^†^	-0.02^†^	

^†^All *F*_*ST*_ values are significant at P = 0.05, excepting values between three wild quail populations.

### Genetic relationships among quail populations inferred by microsatellite markers

**Fig 2:**
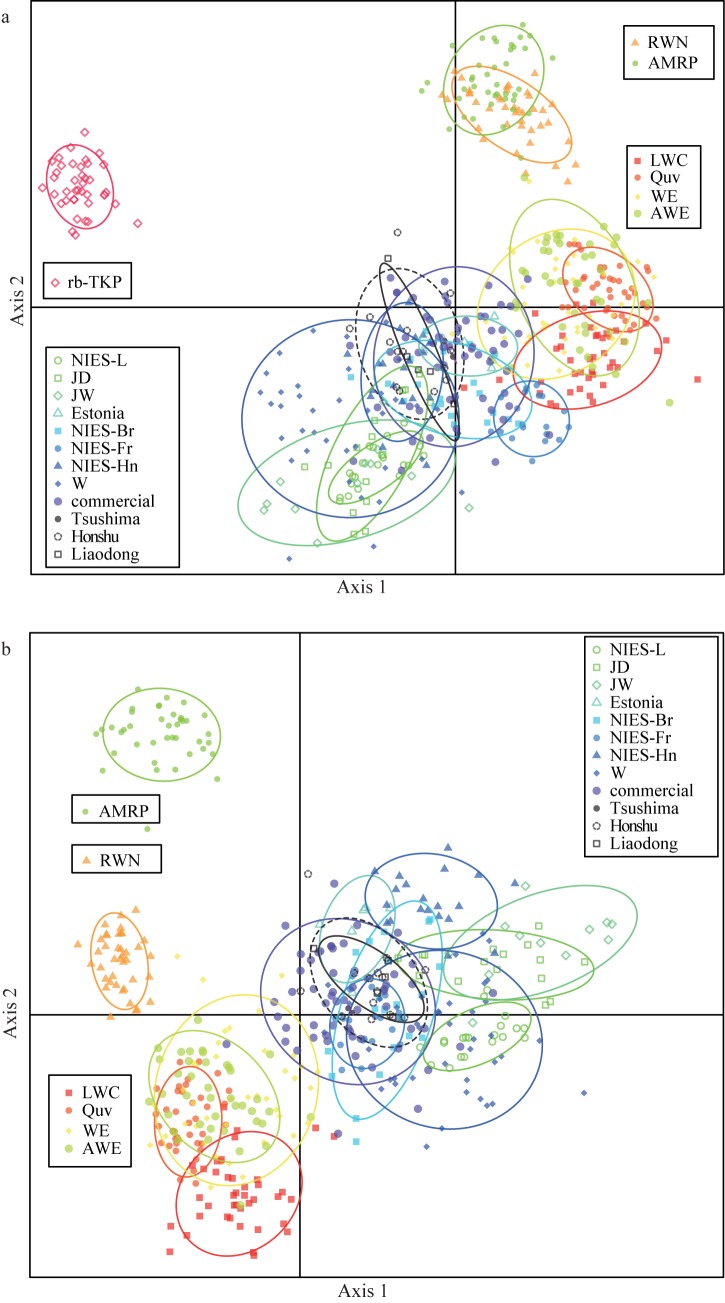
Discriminant analysis of principal components (DAPC) of 19 quail populations constructed using 23 microsatellite markers. (a) DAPC using 19 populations. The populations were subdivided into three groups; a distinct cluster of rb-TKP alone, a cluster of AMRP and RWN, and a group consisting of the other 16 populations. (b) DAPC using 18 populations without rb-TKP. When rb-TKP was excluded, AMRP and RWN were plotted independently as separate clusters. LWC, Quv, WE, and AWE were clustered into the same group. The other laboratory lines, commercial line, and wild quail were grouped into a single cluster by being separated from the cluster of LWC, Quv, WE, and AWE. Each population group is enclosed by an ellipse, which covered 80% of the distribution of individuals of each population.

In the DAPC plot, a laboratory line, rb-TKP, was placed as a separate cluster independently from the other 18 populations (Fig 2A). To define genetic differences among the other 18 quail populations, we performed DAPC without rb-TKP. The result showed that AMRP and RWN were each separated into a different cluster (Fig 2B). The four laboratory lines (LWC, Quv, WE, and AWE) were clustered independently into one group. The other 12 populations including two laboratory lines (NIES-L and W), six meat-type quail lines (JD, JW, Estonia, NIES-Br, NIES-Fr, and NIES-Hn), and commercial and wild quail, were clustered into a single separate group, even though several individuals of WE and the commercial population overlapped.

**Fig 3:**
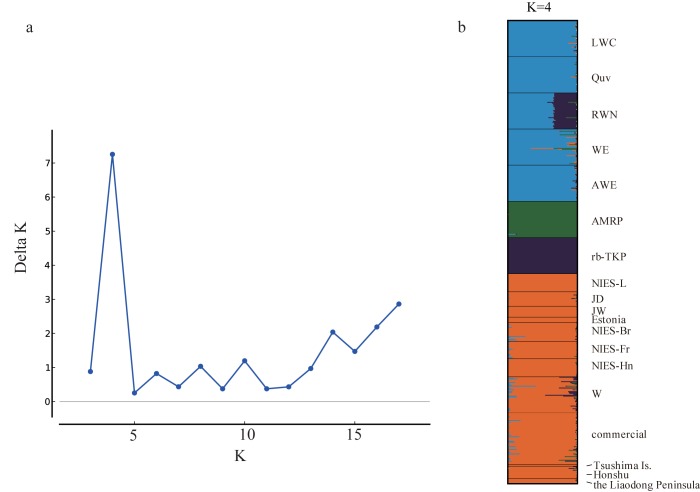
Bayesian clustering of 19 quail populations. (a) Delta K values at K = 2 to K = 18. (b) Group membership of 19 quail populations that can be subdivided into four clusters, which are highlighted by different colors.

**Fig 4:**
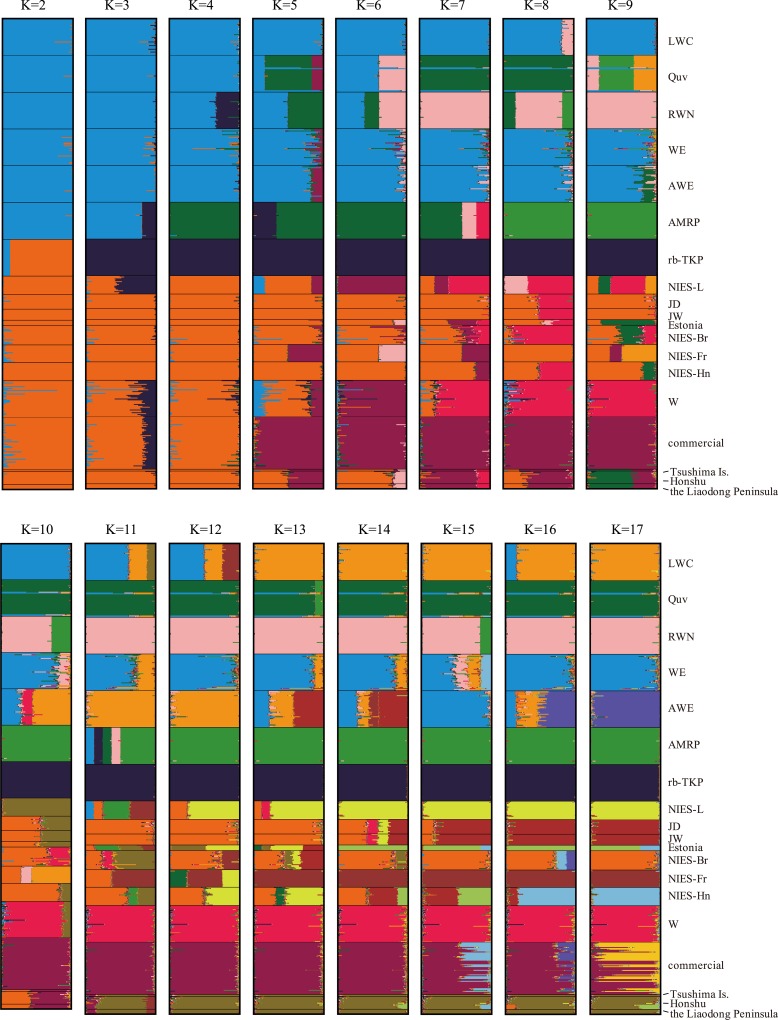
Hierarchical change of group memberships in Bayesian clustering of 19 quail populations. Bayesian clustering patterns at K = 2 to K = 17 are shown.

The Bayesian clustering analysis suggested that the optimal number of clusters was four (K = 4, delta K = 7.26) for our dataset of 19 populations (Fig 3A). Five laboratory quail lines, LWC, Quv, RWN, WE, and AWE, were assigned to one cluster (Microsat-Group1), whereas AMRP was assigned to another cluster in which Quv and RWN showed partial membership (Fig 3B). The rb-TKP line formed the third cluster alone. The other 10 populations were assigned to the fourth cluster (Microsat-Group2), consisting of two laboratory lines established in Japan (NIES-L and W), six meat-type quail lines (JD, JW, Estonia, NIES-Br, NIES-Fr, and NIES-Hn), and commercial and wild quail populations. The hierarchical changes in clustering patterns from K = 2 to K = 17 are shown in Fig 4. In the Bayesian clustering analysis assuming K = 2, 19 populations were divided into two groups; one cluster was composed of Microsat-Group1 and AMRP, and the other groups each consisted of rb-TKP and Microsat-Group2. At K = 3 and K = 4, rb-TKP and AMRP were isolated from the other populations, and rb-TKP consistently constituted an independent cluster for K = 4 to K = 17. Quv and RWN were separated from Microsat-Group1 (LWC, WE, and AWE) at K = 5, and LWC, WE, and AWE were separated at K = 11 and K = 13. Estonia and NIES-Fr were independent of the other four meat-type lines (JD, JW, NIES-Br, and NIES-Hn) at K = 8 and 9.

## Discussion

### Two genetic sources of domestic Japanese quail inferred from the mitochondrial D-loop region

The molecular phylogenetic tree and network of mtDNA D-loop sequences revealed the presence of two clades, Dloop-Group1 and Dloop-Group2, within domestic Japanese quail populations. The wide distribution of JqD1 of Dloop-Group1 suggests that the haplotype recently spread among domestic quail populations around the world. JqD1 was also observed for wild Japanese quail from Honshu in Japan and Liaodong Peninsula in China. In addition, our results suggest that Dloop-Group2 is older than Dloop-Group1 because two haplotypes, F5 and H63, in Dloop-Group2 are considered older than other haplotypes of Japanese quail in European countries [18,19]. F5 is the ancestral haplotype among nine haplotypes of Japanese quail (F1W1, W6, F2−F8) found for farm and wild-captured quail in Spain in a previous study [19]. H63 is also an ancestral haplotype found in wild quail in Mongolia [18]. Meanwhile, H1, which belonged to Dloop-Group1 in this study, is one of the most recent haplotypes based on a phylogenetic tree in a previous study [18]. These results collectively suggest that Dloop-Group2 is a group of haplotypes that expanded in domestic Japanese quail populations earlier than haplotypes in Dloop-Group1. Even though we could not estimate the time of divergence between the two clades due to the small number of substitution sites within the D-loop region examined, our results suggest a relatively recent divergence of the two groups.

The mtDNA diversity was previously investigated for 14 laboratory lines of Japanese quail, including LWC, AMRP, Quv, RWN, and rb-TKP, which were examined in this study, using a restriction fragment length polymorphism (RFLP) analysis [23]. Among 15 restriction endonucleases, *Bam*HI-RFLP revealed that the 14 lines were separated into two groups: one that contained Quv and RWN and the other that included LWC, AMRP, and rb-TKP. This was comparable to the results obtained in this study, except for AMRP. Shen et al. [23] suggested that modern domestic populations of Japanese quail were derived from two different groups of wild Japanese quail. However, our microsatellite data did not confirm their result because no distinct genetic difference was found among wild quail populations collected from three different geographic areas. More samples of wild individuals need to be examined for elucidating whether genetic diversity among wild Japanese quail populations caused genetic differences among domestic populations.

### Genetic divergence and breeding histories of domestic Japanese quail

Genetic clustering analysis using 23 microsatellite markers revealed that 16 domestic quail populations are subdivided into four clusters of populations. Microsat-Group1 consisted of WE and four WE-derived laboratory lines (LWC, Quv, RWN, and AWE) [10,23,50–52], and Microsat-Group2 comprised NIES-L, JD, JW, Estonia, NIES-Br, NIES-Fr, NIES-Hn, W, and commercial and wild populations. AMRP and rb-TKP each constituted a separate cluster. This result is well concordant with the breeding histories of the quail populations [10,23–25,50–57] (Table 4, S1 Fig).

**Table 4:** A priori and posteriori hypotheses for source populations of domestic quail populations inferred from their breeding histories and genetic groups in mtDNA D-loop haplotypes and microsatellite markers.

Line and Population		A priori hypothetical origin inferred from breeding history	Genetic group		A posteriori hypothetical origin inferred from genotypes
			Haplogroup of mtDNA (haplotype)	Genetic cluster of microsatellite markers	
LWC		pre-war/post-war	Dloop-Group1 (JqD1)	Microsat-Group1	pre-war/post-war
Quv		pre-war/post-war	Dloop-Group2 (JqD2)	Microsat-Group1	pre-war
RWN		pre-war/post-war	Dloop-Group2 (JqD3, D5)	Microsat-Group1	pre-war
WE		pre-war/post-war	Dloop-Group1 (JqD1)	Microsat-Group1	pre-war/post-war
			Dloop-Group2 (JqD2, JqD3)		
AWE		pre-war/post-war	Dloop-Group1 (JqD1)	Microsat-Group1	pre-war/post-war
			Dloop-Group2 (JqD2)		
AMRP		pre-war	Dloop-Group2 (JqD2)	AMRP	pre-war
rb-TKP		post-war	Dloop-Group1 (JqD1)	rb-TKP	post-war
NIES-L		post-war	Dloop-Group1 (JqD1)	Microsat-Group2	post-war
JD		post-war	Dloop-Group1 (JqD1)	Microsat-Group2	post-war
JW		post-war	Dloop-Group1 (JqD1)	Microsat-Group2	post-war
Estonia		post-war	Dloop-Group2 (JqD3)	Microsat-Group2	pre-war/post-war
NIES-Br		post-war	Dloop-Group1 (JqD1)	Microsat-Group2	post-war
NIES-Fr		post-war	Dloop-Group2 (JqD3)	Microsat-Group2	pre-war/post-war
NIES-Hn		post-war	Dloop-Group1 (JqD1)	Microsat-Group2	post-war
W			Dloop-Group1 (JqD1)	Microsat-Group2	-
			Dloop-Group2 (JqD2, JqD5)		
commercial		post-war	Dloop-Group1 (JqD1, JqD4)	Microsat-Group2	pre-war/post-war
			Dloop-Group2 (JqD2, JqD3, JqD6)		
wild	Tsushima Is.		Dloop-Group2 (JqD2, JqW3)	Microsat-Group2	-
	Honshu		Dloop-Group1(JqD1)	Microsat-Group2	-
			Dloop-Group2(JqW1, JqW2)		
	the Liaodong Peninsula		Dloop-Group1 (JqD1)	Microsat-Group2	-
			Dloop-Group2 (JqD2)		

The AMRP line originated from a mutant animal with the “*panda*” plumage coloration [53,58,59], which was purchased from a Japanese farm in 1966 and maintained as a closed colony at the Nippon Institute for Biological Science [53]. The *panda* mutation appears in Japanese literature published in 1918, which records that the mutant had been maintained as a pet animal by fanciers [54]. The older type of D-loop haplotype (JqD2 in Dloop-Group2) in AMRP and its genetically distinct position in a genetic clustering analysis indicate that this line derived from the pre-war population.

We consider the genetic background of WE and four WE-derived laboratory lines to be derived from both the pre-war and post-war populations, because the WE line originated from the “CE” line, which was established at the Nippon Institute for Biological Science in Japan in 1966 by the crossbreeding of Japanese commercial quail (post-war population) with a laboratory line imported from the United States (pre-war population) (Mizutani M, personal communication). The mixture of populations from different origins was found for their D-loop haplotypes: LWC, WE, and AWE lines have the JqD1 haplotype of Dloop-Group1, which is considered to be of post-war origin, while Quv and RWN have the JqD3 haplotype of Dloop-Group2, which may have derived from the pre-war population. Genetic differences between the two groups also become apparent by the DAPC and the Bayesian clustering, and Quv and RWN are more closely related to AMRP. This suggests that the genetic background of the WE and WE-derived lines originated from both post-war and pre-war populations.

The meat-type lines clustered together in Microsat-Group2 with NIES-L and commercial quail. The body weights of NIES-Br and NIES-Hn quail are approximately two-fold higher than those of common laboratory and commercial quail. For example, average body weights of adult males and females of NIES-Br are 260.3 g and 289.5 g, respectively, while NIES-L males are 122.9 g and females are 148.0 g (Avian Bioscience Research Center, Nagoya University, http://www.agr.nagoya-u.ac.jp/~nbrp/en/index.html). The close genetic relationship among these lines indicates that most of existing domestic populations were recently distributed worldwide from the post-war founder population [10,12–14], although the biological features are quite different among populations. The Estonia and NIES-Fr lines retained the haplotypes of Dloop-Group2 (JqD2 and JqD3), which were possibly derived from the pre-war population, and these lines are apparently distinct from the other lines clustered into Microsat-Group2 in the pairwise *F*_*ST*_ network and the Bayesian clustering at K = 8. These results suggest that some quail populations clustered into Microsat-Group2, such as Estonia and NIES-Fr, initially retained the genetic background of both pre-war and post-war populations, and the genetic contribution of the pre-war population decreased over time during some selective breeding process, such as backcrossing with quail derived from the post-war population.

The rb-TKP line was established independently from the other laboratory lines and maintained at Takeda Pharmaceutical Co. Ltd. in Japan since 1983 [23]. The genetic distinctiveness of this line shown in the DAPC plot and the Bayesian clustering seems to be attributed to a high degree of genetic homogenization in this line, as evidenced by the low expected heterozygosity and allelic richness. The rb-TKP has the haplotypes of Dloop-Group1 and was clustered into Microsat-Group2 by the Bayesian clustering analysis assuming K = 2, which suggests that this line retains genetic components from the post-war population.

The pairwise *F*_*ST*_ values among 19 quail populations including three wild quail populations were remarkably higher than those of domestic chicken populations in India (*F*_*ST*_ = 0.06−0.14, 0.094 on average, in eight domestic populations) [60], China (*F*_*ST*_ = 0.176−0.302, 0.231 on average, in seven indigenous populations) [61], and Italy (*F*_*ST*_ = 0.03−0.32, 0.180, on average, in six local breeds and four commercial lines) [62]. The high *F*_*ST*_ values in the quail populations used in this study seem to be attributed to their breeding history and the characteristics of microsatellite markers used in this study. Nine of the 15 lines (LWC, Quv, RWN, WE, AWE, AMRP, rb-TKP, NIES-L, and W) were maintained independently as closed colonies for more than twenty years. In this study, microsatellite markers with high allelic polymorphism were selected from more than 100 markers developed in our previous studies [24,25]. These factors are considered to increase the number of alleles and inflate differences in allele frequencies among populations, leading to the high *F*_*ST*_ values.

## Conclusions

We identified two genetic sources of domestic Japanese quail populations with different genetic backgrounds by analyses of mtDNA D-loop sequences and microsatellite markers. One (Dloop-Group2 and Microsat-Group1) may include alleles from the population that was exported to the United States from Japan before World War II (the pre-war population). The other (Dloop-Group1 and Microsat-Group2) was probably derived from limited founder populations that were re-established in Japan after the war (the post-war population), which were subsequently exported from Japan and then rapidly spread around the world. Components of the genetic background of the pre-war population can be found in the present domestic quail population, which is the result of persistent crossbreeding with the post-war population. Further studies with additional samples, including wild Japanese quail, are needed to obtain the detailed genetic characteristics of domestic quail populations in the world.

## Supporting Information

S1 Fig Graphical chart of breeding histories of 16 domestic quail populations used in this study based on previous reports [10,23,55]. Putative pre-war and post-war populations are shown by ellipses and hexagons, respectively. Dotted and solid lines indicate breeding histories before and after establishment (introduction) of quail populations, respectively. Filled diamonds indicate a cross between different populations. Genetic groups based on microsatellite markers are shown on the right side.
(PDF)

S2 Fig The relationships between the number of amplified microsatellite markers and the age of wild quail samples. Tissue samples stored in 70% ethanol are shown by filled orange diamonds and feather shaft samples of stuffed specimens by filled gray diamonds.
(PDF)

S3 Fig Neighbor joining tree of 17 quail populations based on pairwise *F*_*ST*_ distance. (PDF)

S1 Table List of wild Japanese quail used in this study and their individual information. (XLSX)

S2 Table Origins and genetic characteristics of Japanese quail lines and populations used in this study. (XLSX)

S3 Table Accession numbers of mitochondrial D-loop haplotypes of Japanese quail lines and populations examined in this study. (XLSX)

S4 Table Variable sites in the 278 (279)-bp D-loop region of nine haplotypes. (XLSX)

S5 Table The number of individuals for which each microsatellite marker was amplified. (XLSX)

S6 Table The chi-square test for Hardy-Weinberg equilibrium and fixation index (*F*). (XLSX)

S1 File Microsatellite genotypes for Japanese quail samples used in this study. (XLSX)
