# Supplementary figures and images for: Genetic Divergence in Domestic Japanese Quail Inferred from Mitochondrial DNA D-Loop and Microsatellite Markers
Source: PLoS One. 2017 Jan 20;12(1):e0169978. doi: 10.1371/journal.pone.0169978 (PMC5249226; doi:10.1371/journal.pone.0169978)

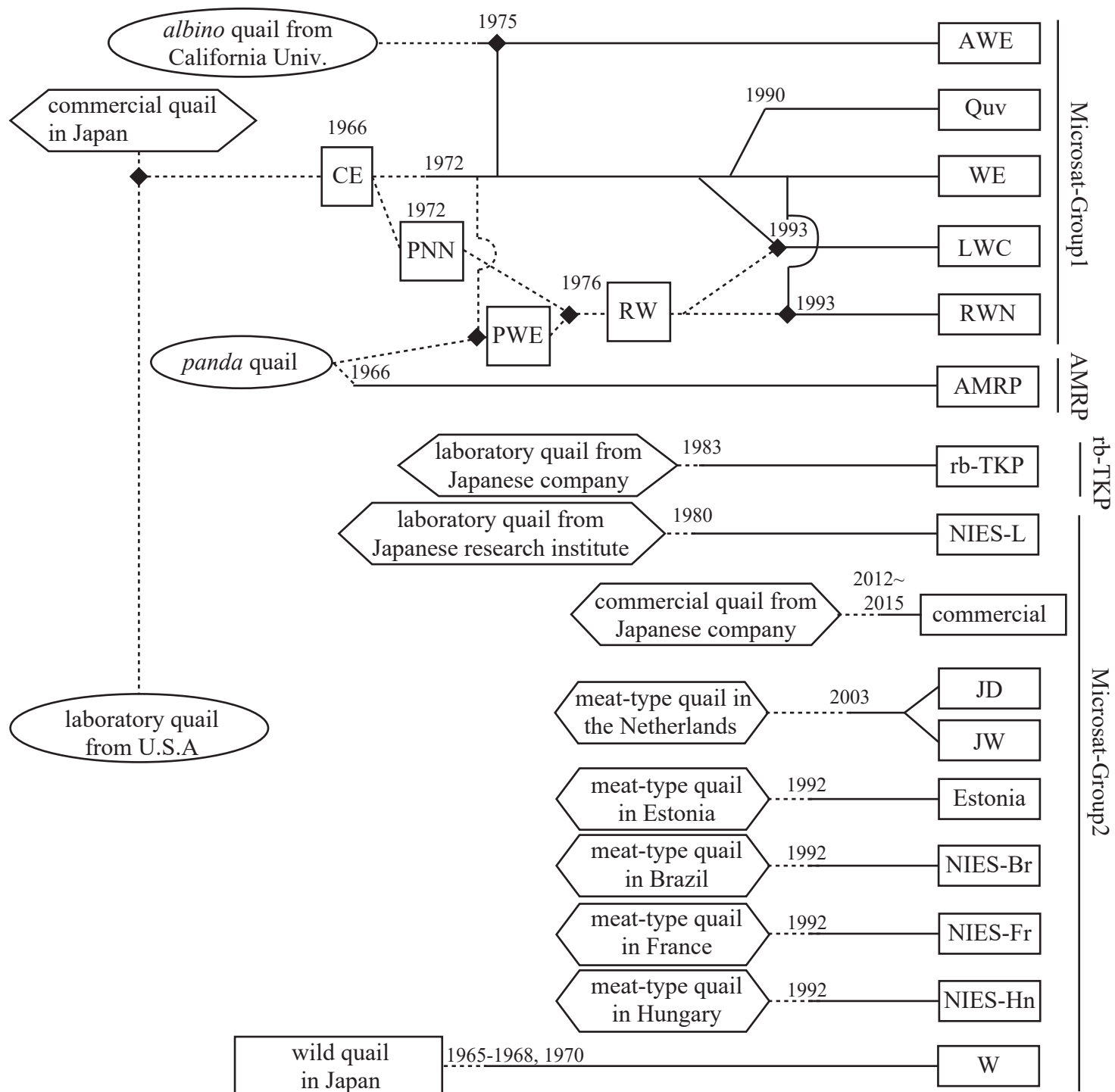

Supplement: S1 Fig — Putative pre-war and post-war populations are shown by ellipses and hexagons, respectively. Dotted and solid lines indicate breeding histories before and after establishment (introduction) of quail populations, respectively. Filled diamonds indicate a cross between different populations. Genetic groups based on microsatellite markers are shown on the right side. (PDF) [file pone.0169978.s001.pdf]

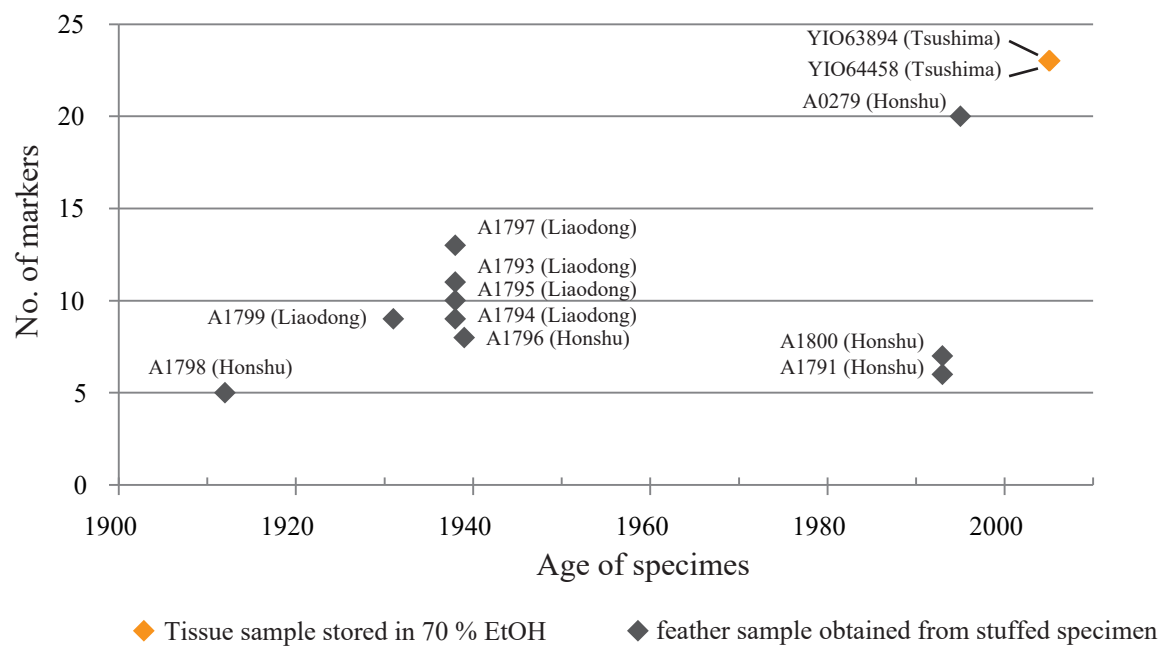

Supplement: S2 Fig — Tissue samples stored in 70% ethanol are shown by filled orange diamonds and feather shaft samples of stuffed specimens by filled gray diamonds. (PDF) [file pone.0169978.s002.pdf]

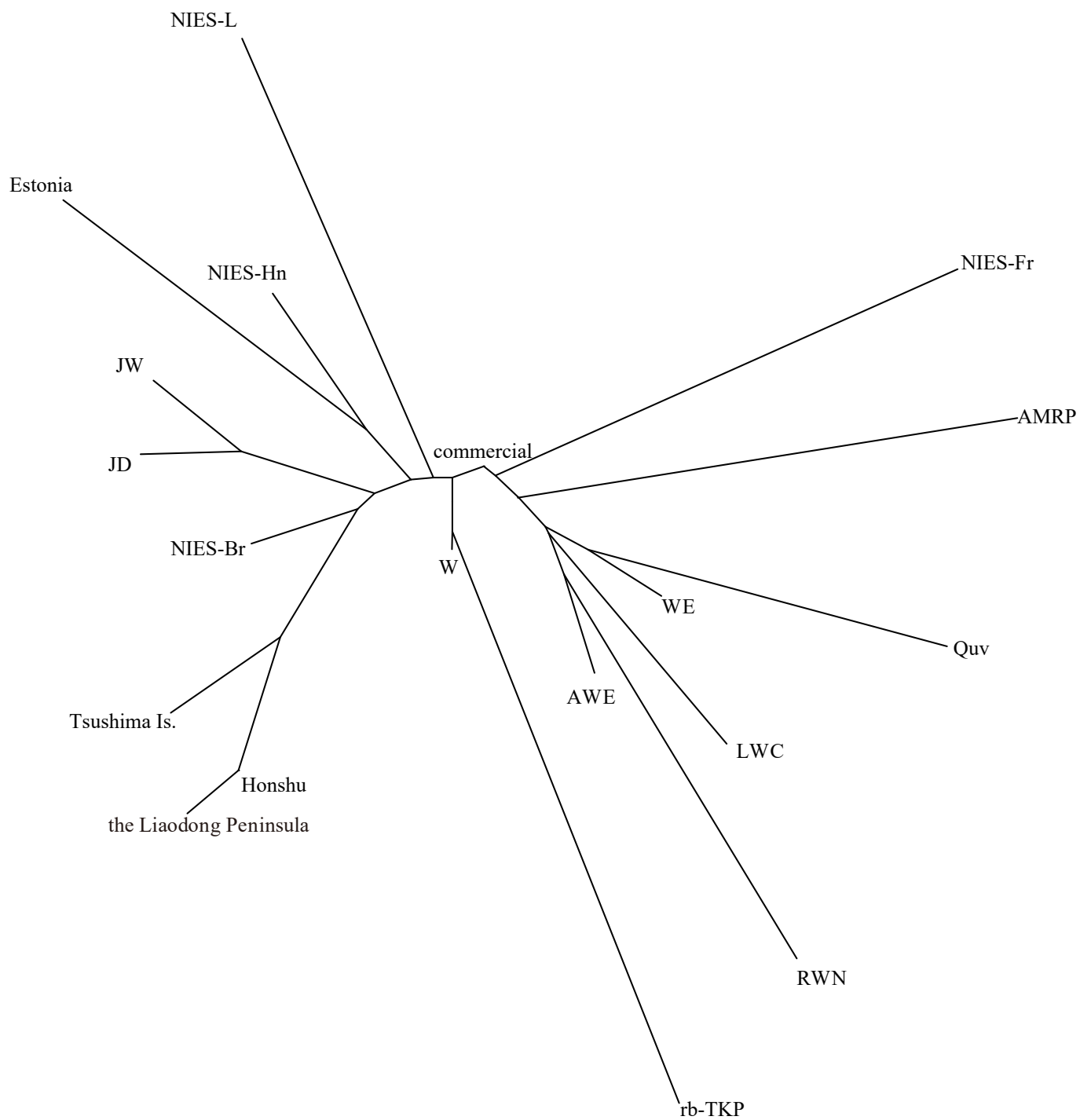

Supplement: S3 Fig — (PDF) [file pone.0169978.s003.pdf]
